# Genome-Wide Analysis of Human MicroRNA Stability

**DOI:** 10.1155/2013/368975

**Published:** 2013-09-28

**Authors:** Yang Li, Zhixin Li, Shixin Zhou, Jinhua Wen, Bin Geng, Jichun Yang, Qinghua Cui

**Affiliations:** ^1^Department of Cell Biology, Stem Cell Research Center, Peking University School of Basic Medical Sciences, 38 Xueyuan Road, Beijing 100191, China; ^2^Department of Integrated Chinese and Western Medicine, Peking University School of Basic Medical Sciences, 38 Xueyuan Road, Beijing 100191, China; ^3^Department of Physiology and Pathophysiology, Peking University School of Basic Medical Sciences, 38 Xueyuan Road, Beijing 100191, China; ^4^MOE Key Lab of Molecular Cardiovascular Science, Peking University, 38 Xueyuan Road, Beijing 100191, China; ^5^Department of Biomedical Informatics, Peking University School of Basic Medical Sciences, 38 Xueyuan Road, Beijing 100191, China

## Abstract

Increasing studies have shown that microRNA (miRNA) stability plays important roles in physiology. However, the global picture of miRNA stability remains largely unknown. Here, we had analyzed genome-wide miRNA stability across 10 diverse cell types using miRNA arrays. We found that miRNA stability shows high dynamics and diversity both within individual cells and across cell types. Strikingly, we observed a negative correlation between miRNA stability and miRNA expression level, which is different from current findings on other biological molecules such as proteins and mRNAs that show positive and not negative correlations between stability and expression level. This finding indicates that miRNA has a distinct action mode, which we called “rapid production, rapid turnover; slow production, slow turnover.” This mode further suggests that high expression miRNAs normally degrade fast and may endow the cell with special properties that facilitate cellular status-transition. Moreover, we revealed that the stability of miRNAs is affected by cohorts of factors that include miRNA targets, transcription factors, nucleotide content, evolution, associated disease, and environmental factors. Together, our results provided an extensive description of the global landscape, dynamics, and distinct mode of human miRNA stability, which provide help in investigating their functions in physiology and pathophysiology.

## 1. Introduction

MicroRNAs (miRNAs) are one class of endogenous small noncoding RNAs, which mainly regulate gene expression at the posttranscriptional level by binding to the 3′UTRs of the target mRNAs through base pairing, resulting in cleavage or translation inhibition of target mRNA [1]. To date, more than 1500 miRNA genes (miRBase [2], Release 18) have been identified in the human genome. On average, one miRNA is predicted to regulate hundreds of target genes, and it is also estimated that more than one-third of human genome genes may be regulated by miRNAs. In theory, it is expected that RNA molecules (both coding and noncoding) could bind with miRNAs, and therefore, miRNAs are hypothesized as a bridge linking the world of RNAs [3]. Clearly, miRNAs tend to be one class of critically important molecules. Indeed, increasing evidence has shown that miRNAs play key roles in a broad range of biological functions such as metabolism, proliferation, development, differentiation [4, 5], apoptosis [6], immune response [7], cell communication [8], and environmental adaptation [9]. miRNAs have been shown to be involved in a wide spectrum of diseases [10, 11]. 

For a biological molecule, it is critically important to maintain a suitable abundance in specific time, space, and cellular condition. This is also true for miRNAs. Given their important functions, deregulated miRNA abundance will have severe effects on their downstream pathways and therefore implicate in the pathogenesis of diseases. Abnormal miRNA abundance has been reported to be associated with a number of human diseases according to the records of the Human microRNA Disease Database (HMDD) [11]. The abundance of a biological molecule is mainly determined by the rate of its transcription as well as the rate of its degradation (stability). In recent years, a number of studies had systematically analyzed global stability of mRNAs [12–18], proteins [19], and long noncoding RNAs (lncRNAs) [20, 21] using high-throughput techniques, for example, microarray. Moreover, further analysis of the global stability of biological molecules has revealed that molecule stability does contribute significantly to their abundance [21]. More importantly, a number of important biological insights have been revealed. For example, a molecule stability is closely related to its physiological function [13–15]. Stability of protein, mRNA, and noncoding transcript is positively correlated with expression level [19, 20]. Basically, the stability of housekeeping genes is high, whereas the stability of transcription factors is low [20–22]. More recently, emerging studies have reported important roles of miRNA stability in physiology and revealed some factors that affect miRNA stability [23]. For example, the degradation of miRNAs can be mediated by the 3′→5′ exonuclease SDN1 in *Arabidopsis *[24] and by the 5′→3′ exoribonuclease XRN-2 in *Caenorhabditis elegans* [25]. Hwang et al. observed that human miR-29b has a high stability in mitotic cells but is degraded rapidly in cycling cells [26]. The authors further revealed that the reason is that miR-29b is predominant within the nucleus, which prevents miRNA-29b from being rapidly degraded [26]. Gantier et al. identified the global miRNA stability of mouse embryonic fibroblasts and found that miRNA stability was mostly affected by cell division. They also found that miRNAs are more stable than mRNAs [27]. Recent studies showed that although miRNAs normally have a long life span, they also degrade rapidly in some specific conditions [28, 29]. For example, the rapid turnover of miR-183/96/182 cluster, miR-204, and miR-211 plays a critical role in the physiology of retinal light regulation. Moreover, the rapid turnover is shown to be dependent on neuronal activity [28], suggesting that abnormal miRNA stability might take part in pathophysiology. Overall, although the above studies greatly improved our understanding on miRNA stability, the global landscape of human miRNA stability remains largely unknown and needs to be explored emergently in a genome-wide analysis.

In the current study, we had performed a genome-wide analysis of miRNA stability in 10 human cell lines (see Table 1 for details) that covered a broad range of cell types and represented a diversity of derived tissues using custom microarrays that integrate ~1900 human miRNAs (miRBase, Release 18) and ~150 virus miRNAs. We found that miRNA stability shows high dynamics and diversity both within cells and across cells. Although some miRNAs are stable, there are some miRNAs with rapid turnover. Different cell types show significantly different miRNA stability, suggesting that miRNA stability is highly cell selective and may play roles in defining cellular identity and function. Strikingly, we revealed a distinct picture of miRNA stability; that is, miRNA stability shows a significant negative correlation with miRNA expression level, which is opposite to current findings on other biological molecules such as protein, mRNA, and noncoding transcript. This suggests that miRNAs show a special mode of actions, which may be dependent on its specific manner of negative gene regulation. In addition, we revealed a number of factors that affect miRNA stability. 

## 2. Materials and Methods

### 2.1. Cell Culture and RNA Extraction

HEK293, U251, BGC-823, Panc-1, MCF-7, A549, PC-3 M-IE8, and T/G HA-VSMC cells were maintained in DMEM (HyClone) supplemented with 10% fetal bovine serum (Gibco). Human embryonic stem cells (H9) were cultured on Matrigel (Becton Dickinson) as described by Xu et al. [30] in Knockout DMEM (Gibco) supplemented with 20% serum replacement (Gibco), 0.1 mM *β*-mercaptoethanol (Gibco), 1% nonessential amino acids (Hyclone), 2 mM glutamine, and 8 ng/mL basic FGF (Peprotech). Human neural stem cells (NSCs) were cultured as described by Li et al [31] in DMEM/F12 supplemented with 2% fetal bovine serum (Gibco), B27 supplement (Gibco), 20 ng/mL EGF (Peprotech), and 20 ng/mL basic FGF (Peprotech). For stability experiments, we performed the experiments based on the protocol of Krol et al. [28]. Cells were grown to 70% confluency before RNA polymerase activity was blocked with 10 *μ*g/mL actinomycin D (Sigma) in DMSO. Control cells were treated with DMSO alone. Transcriptional inhibition was conducted for 3h, with cells harvested at time 0h and after 3h. Cells were collected by trypsinization, and total RNA was extracted using miRNeasy columns (QIAGEN) and treated with DNase. 

### 2.2. Calculation of miRNA Stability

To identify the stability of miRNAs, we inhibited transcription of ten types of human cells (Table 1) with actinomycin D and measured RNA levels at time points of 0h and 3h. We next performed genome-wide analysis of miRNA stability based on the 7th generation of Exiqon miRCURY LNA expression miRNA array. 

*(1) miRNA Microarrays.* The 7th generation of Exiqon miRCURY LNA expression miRNA array contains all human miRNAs (~1900) annotated in miRBase 18.0 as well as all viral miRNAs (~150) related to human. 

*(2) RNA Extraction.* Total RNA was isolated using TRIzol (Invitrogen) and miRNeasy mini kit (QIAGEN) according to manufacturer's instructions, which efficiently recovered all RNA species, including miRNAs. RNA quality and quantity were measured by using nanodrop spectrophotometer (ND-1000, Nanodrop Technologies), and RNA integrity was determined by gel electrophoresis. 

*(3) RNA Labeling.* After RNA isolation from the samples, the miRCURY Hy3/Hy5 power labeling kit (Exiqon, Vedbaek, Denmark) was used according to the manufacturer's guidelines for miRNA labelling. One microgram of each sample was 3′-end-labeled with Hy3 fluorescent label, using T4 RNA ligase by the following procedure: RNA in 2.0 *μ*L of water was combined with 1.0 *μ*L of CIP buffer and CIP (Exiqon). The mixture was incubated for 30 min at 37°C and was terminated by incubation for 5 min at 95°C. Then, 3.0 *μ*L of labeling buffer, 1.5 *μ*L of fluorescent label (Hy3), 2.0 *μ*L of DMSO, and 2.0 *μ*L of labeling enzyme were added to the mixture. The labeling reaction was incubated for 1 h at 16°C, and terminated by incubation for 15 min at 65°C.

*(4) Array Hybridization.* After stopping the labeling procedure, the Hy3-labeled samples were hybridized on the miRCURYTM LNA Array (v.18.0) (Exiqon) according to array manual. The total 25 *μ*L mixture from Hy3-labeled samples with 25 *μ*L hybridization buffer was first denatured for 2 min at 95°C, incubated on ice for 2 min, and then hybridized to the microarray for 16–20 h at 56°C in a 12-bay hybridization systems (Hybridization System-Nimblegen Systems, Inc., Madison, WI, USA), which provides an active mixing action and constant incubation temperature to improve hybridization uniformity and enhance signal. Following hybridization, the slides were achieved, washed several times using Wash Buffer Kit (Exiqon), and finally dried by centrifugation for 5 min at 400 rpm. Then, the slides were scanned using the Axon GenePix 4000B microarray scanner (Axon Instruments, Foster City, CA).

*(5) Data Analysis.* Scanned images were then imported into GenePix Pro 6.0 software (Axon) for grid alignment and data extraction. miRNAs in which foreground-background intensities smaller than 30 were considered to be not reliable and then were filtered. miRNA expression data was then normalized relative to the expression level of U6. We defined the miRNA stability index (MSI) of a miRNA as the fold of its expression level at 3h to the level at 0h. Because of microarray technique or other unknown factors, some probes show higher expression level at 3h than at 0h. We found that these probes did not affect the main results of this study. We therefore did not consider these probes in this study. We then determined the MSI for all miRNAs across ten cell types. The raw miRNA expression profile data, the normalized miRNA expression profile data, and the MSI data are publicly available at the website: http://202.38.126.151/hmdd/tools/msi.html. 

### 2.3. Data of miRNAs

We obtained human miRNA sequence data from miRBase [2] (Release 18.0) and calculated the nucleotide content and miRNA length based on the sequences. We obtained experimentally supported miRNA target data from TarBase [32] (version 5.0) and miRRecords [33] (version 3.0). We obtained the experimentally TF-miRNA regulatory pairs from TransmiR [34]. We downloaded the miRNA family data from miRBase. Using the method presented by Wang et al. [35], we assigned miRNAs into five groups according to their conservation. Group 1 represents the most conserved miRNAs, which are conserved in the invertebrates. Group 2 to 4 are miRNAs that are conserved in vertebrates, mammals, and primates, respectively. Group 5 contains miRNAs that do not have family members in other species according to current miRNA family data (human specific miRNAs). We obtained intronic miRNAs and the host genes from miRBase. We downloaded the data of miRNA disease spectrum width (DSW) from the Human microRNA Disease Database (HMDD) [11]. DSW was defined as the fraction of the number of diseases associated with a miRNA to that of whole diseases associated with all miRNAs [36]. DSW of a miRNA is defined to measure its importance or chance in the development and progression of human diseases. We obtained the miRNAs that respond to EFs in the MCF-7 cell from the miREnvironment database [37]. 

### 2.4. Data Analysis and Statistical Computing

We used pajek to visualize the network of cell-cell relationship generated by correlation analysis. We calculated the distances between any two cells based on their correlation coefficients and mapped the ten cells onto a 2-dimensional Euclidean space using the technique of multidimensional scaling (MDS). We used TAM [38] (version 2) to perform miRNA functional enrichment analysis. Clustering analysis was performed using the software of Cluster and the visualization of clustering result was performed using the software of TreeView [39]. Statistical computations and tests were performed using R, a statistical computing language (http://www.r-project.org/), and the correlations were calculated using Spearman's correlation, a nonparametric method.

## 3. Results and Discussion

### 3.1. Genome-Wide Identification of miRNA Stability

To explore the stability of miRNAs, we inhibited transcription of ten types of human cells (Table 1) with actinomycin D [28] and measured miRNA levels at time points of 0h and 3h. It is a common difficult problem to select a suitable time point for the detection of miRNA expression level in the analysis of molecular stability. A short time means that the expression levels of stable miRNAs may not change enough, whereas a long time means that the unstable miRNAs may totally disappear. Given that a time point of 3h seems good in the investigation of abundance changes of both stable and unstable retinal miRNAs in mammal light adaptation process [28], we selected 3h in this study. Although it is impossible to be a perfect time point for all cell types, 3h is relatively suitable to distinguish stable and unstable miRNAs. To obtain a global and comprehensive landscape of miRNA stability, we selected 10 human cell lines that cover diverse cell types and represent a wide range of derived tissues, including lung, stomach, embryonic kidney, blastocyst, prostate, muscle, breast, embryonic brain, pancreas, and brain. These diverse cell types allow us to not only investigate the diversity of miRNA stability within individual cells but also explore the dynamics of miRNA stability across various cell types. We next performed genome-wide analysis of miRNA stability based on the 7th generation of Exiqon miRCURY LNA expression miRNA array, which contains all human miRNAs (~1900) annotated in miRBase 18.0 as well as all viral miRNAs (~150) related to human. miRNAs in which foreground-background intensities are smaller than 30 were considered to be not reliable and then were filtered. miRNA expression data was then normalized relative to the expression level of U6. We defined the miRNA stability index (MSI) of a miRNA as the fold of its expression level at 3h after actinomycin D treatment to that at 0h. We then determined the MSI for all miRNAs across the ten cells according to this rule. 

**Figure 1:**
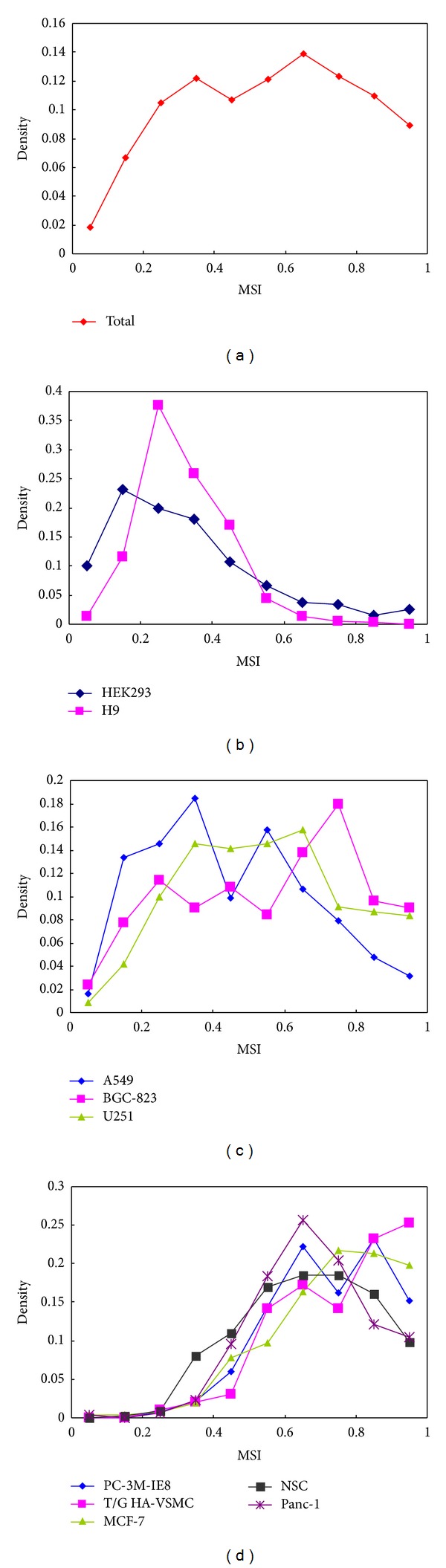
Distribution of miRNA stability (miRNA stability index, MSI) in total cells (a), in the HEK293 and H9 cells (b), in the A549, BGC-823, and U251 cells (c), and in other cells (d).

To examine a global stability of miRNAs and make a comparison across different cell types, we first analyzed the distribution of miRNA stability. The total miRNA stability in the ten cell types shows a relatively balanced distribution with two peaks (Figure 1(a)). One peak is around 0.35 (MSI), and the other is around 0.65 (MSI), suggesting that the global miRNA stability spans a broad range and shows high dynamics and diversity. In spite of some stable miRNAs, there are a number of unstable miRNAs (Figure 1(a)). Unlike the two-peak distribution of global miRNA stability in our study, previous studies have reported that the distribution of (both protein-coding and long noncoding) RNA stability shows a one-peak distribution [20, 21], which skewed to low stability. This difference may be resulted from that the global miRNA stability is a merge of miRNA stability from ten cell types and different cell types may have different distributions of miRNA stability. Indeed, the ten cell types show significantly different distributions of miRNA stability (Figures 1(b), 1(c), and 1(d)). HEK293 and H9 cells, two embryo-related cells, show one-peak distribution of miRNA stability skewed to low stability (Figure 1(b)), whereas PC-3 M-IE8, T/G HA-VSMC, MCF-7, NSC, and Panc-1 cells show one-peak distribution skewed to high stability (Figure 1(d)). In contrast, A549, BGC-823, and U251 cells show relatively balanced two-peak distributions (Figure 1(c)). These results suggested that different cell types have distinct profile of miRNA stability. These results also suggested that miRNA stability may have important roles in defining cellular identity and function. 

**Figure 2:**
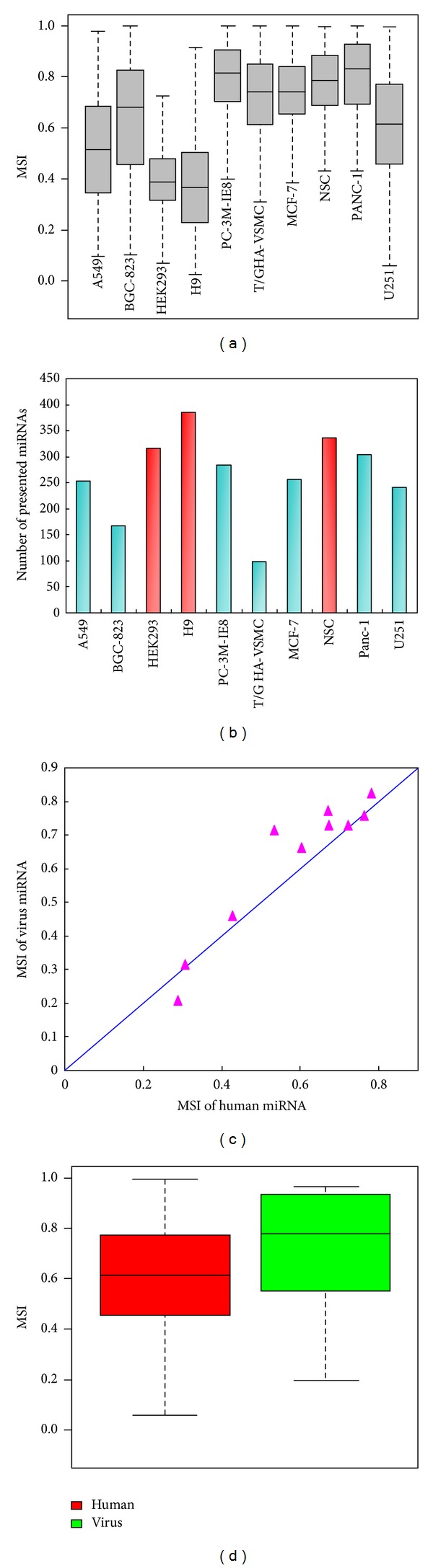
Comparison of miRNA stability among cell types. (a) miRNA stability (miRNA stability index, MSI) in ten cell types. (b) Numbers of presented miRNAs in ten cell types. (c) Median MSI of human miRNAs and virus miRNAs in ten cell types. (d) MSI comparison of human miRNAs and virus miRNAs in the U251 cell.

The differences in the distributions of miRNA stability among various cell lines indicated that different cell types have different miRNA stability. Statistical comparison of miRNA stabilities among the ten cell types did reveal significant differences among these cells (Figure 2(a), *P* < 2.2 × 10^−16^, Kruskal-Wallis test). H9 and HEK293 cells have significantly lower miRNA stability than other cells (median MSI, 0.30 versus 0.66, *P* < 2.2 × 10^−16^, Wilcoxon test). For example, the stability of 93.5% (361/386) of the presented miRNAs in the human embryonic stem cell (H9) is less than 0.5, whereas only 6.1% (6/99) of the presented miRNAs is less than 0.5 in the human smooth muscle cell (T/G HA-VSMC) (*P* < 2.2 × 10^−16^, Fisher exact test). However, the neural stem cell (NSC), also an embryo-derived cell, shows relatively high miRNA stability. Interestingly, the three embryo-derived cells have more presented miRNAs than other cells (Figure 2(b), *P* = 0.008, Wilcoxon test). Although the exact reasons why different cell types show clearly diverse pattern of miRNA stability remain unknown, we hypothesized that the cell diversity of miRNA stability should be related to the functions and states of the cell. Besides the miRNAs encoded by the human genome (human miRNAs), all types of cells expressed a number of miRNAs encoded by virus genomes (viral miRNAs). Similar to human miRNAs, the stability of viral miRNAs also show obvious diversity among these cells (Figure 2(c)). Moreover, the stability of human miRNAs is highly correlated with that of viral miRNAs across the ten cell lines (*R* = 0.92, *P* = 4.7 × 10^−4^, Figure 2(c)). In addition, viral miRNAs tend to be more stable than human miRNAs (*P* = 0.05, paired *t*-test, Figure 2(c)). For example, the stability of viral miRNAs is significantly greater than that of human miRNAs in the human glioblastoma cell (U251) (Median MSI 0.72 versus 0.53, *P* = 0.05, Wilcoxon test, Figure 2(d)). 

We next investigated the relationships of the ten cell types in the context of miRNA stability. For doing so, we performed correlation analysis between any two cell types (see Table S1 in Supplementary Material available online at http://dx.doi.org/10.1155/2013/368975). To obtain a global view of cell relationships, we draw a network of these cells (Supplementary Figure S1) by taking cells as nodes and linking two cells if the correlation between the stabilities of their miRNAs is significant (*P* ≤ 0.05) and draw a scatter plot (Supplementary Figure S1) using multiple dimensional scaling (MDS), which mapped these cells into a Euclidean space. For the MDS analysis, we constructed the distance matrix based on correlation scores among the samples. Both figures show that miRNA stability could be an inherent characteristic that connects cells. For example, the glioblastoma cell (U251) shows strong correlation with the neural stem cell (NSC) in miRNA stability (*R* = 0.48, *P* < 2.2 × 10^−16^), suggesting that miRNAs in the two cells have similar tendency of miRNA stability changes. 

### 3.2. Relationship between miRNA Stability and miRNA Expression Level

**Figure 3:**
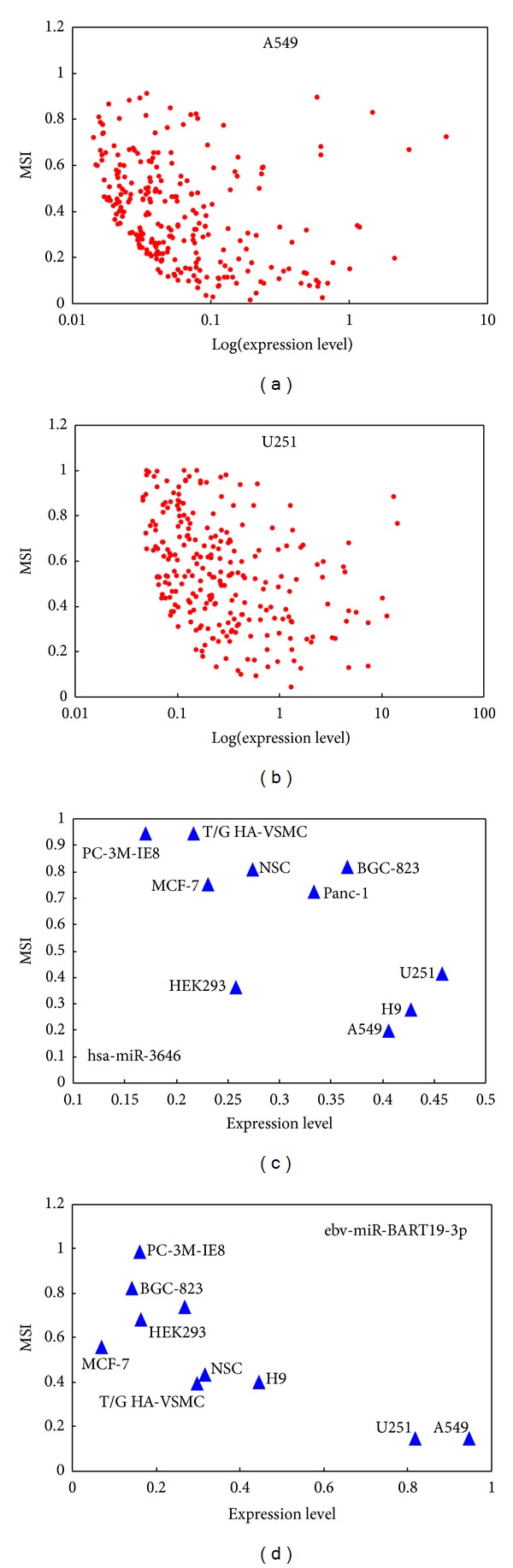
Negative correlation between miRNA stability (miRNA stability index, MSI) and miRNA expression level within the cell A549 (a), within the cell U251 (b), across ten cell types for hsa-miR-3646 (c), and across ten cell types for ebv-miR-BART19-3p (d).

A number of previous studies have revealed that there exists a significant positive correlation between the expression level and the stability of a biological molecule such as protein [19], RNA [20], and noncoding transcript [21]. Basically, highly expressed molecules tend to be stable, whereas lowly expressed molecules tend to have rapid turnover. In the same study by Clark et al. [20], however, no correlation was found between the expression level and stability of lncRNAs, which suggests that not all types of molecules comply with the general rules for the relationship between the stability and expression level. Currently, the relationship between the stability and expression level of miRNAs remains unknown. To address this question, correlation analysis between miRNA stability and miRNA expression level (at 0h) in the ten cell types was performed. Strikingly, the result revealed negative and not positive correlations between miRNA stability and its expression level (Table 1), which is different from other known biological molecules such as protein, mRNA, and noncoding transcript. Nine of the ten cell types show significant negative correlations between miRNA stability and miRNA expression level (Table 1) such as the human lung adenocarcinoma epithelial cell (A549, *R* = −0.42, *P* = 1.37 × 10^−12^, Figure 3(a)) and the human glioblastoma cell (U251, *R* = −0.44, *P* = 2.19 × 10^−13^, Figure 3(b)). Although the correlation in the human embryonic stem cell (H9) is not statistically significant, it also shows an obvious tendency of negative correlation (Table 1). Krol et al. previously reported that neuron-specific miRNAs have rapid turnover in mouse neurons but other miRNAs, for example, miRNAs enriched in glial cells, are stable in these neurons [28]. Because neuron-specific miRNAs normally have relative higher expression level in neurons than many other miRNAs, for example, glial cell enriched miRNAs, the observation by Krol et al. may support our finding that miRNAs with high expression level have rapid turnover and those with low expression level degrade slowly. In addition, by analyzing the changes of miRNA expression levels during the physiological process of light adaptation (LA) to dark adaptation (DA) of mouse retinas, we observed a negative correlation between miRNA stability and miRNA level, although the correlation is not significant (*R* = −0.14, *P* = 0.08) but shows an obvious tendency. This result suggested that the negative correlation between miRNA stability and miRNA level may also exist in vivo. We also searched the GEO and ArrayExpress databases for possible public miRNA stability data but did not find miRNA stability datasets. When more miRNA stability datasets become available in the future, this pattern revealed here would be reinvestigated.

The above results indicated that highly expressed miRNAs tend to have rapid turnover, but lowly expressed miRNAs tend to be stable in a specific cell type. However, for a specific miRNA, the relationship between its stability and its expression level across various cell types remains unknown. Therefore, we further analyzed the correlation between miRNA stability and miRNA level across the ten cell types for single miRNA. The result shows that the relationship keeps unchanged. There are only seven miRNAs that have valid stability value in all the ten cell types. Although some of the correlations are not statistically significant due to limited number of cell types (only ten cell types), the tendency of negative correlation is obvious for all these miRNAs (Supplementary Table S2). For example, significant negative correlation across the ten cell types exists in hsa-miR-3646 (*R* = −0.7, *P* = 0.03, Figure 3(c)) and ebv-miR-BART19-3p (*R* = −0.82, *P* = 0.007, Figure 3(d)). 

Taken together, our findings indicated that miRNA stability is negatively correlated with miRNA expression level both within individual cells and across cell types, which is different from protein (positive correlation), mRNA (positive correlation), noncoding transcript (positive correlation), and lncRNAs (no correlation). These observations revealed a novel relationship between the stability and expression level of a biological molecule, which may present an important action mode of miRNAs, “rapid production, rapid turnover, and slow production, slow turnover.” This mode suggests that highly expressed miRNAs may be mainly involved in status-transition of biological and cellular processes. In order to do tasks of cellular transition, these miRNAs need to be produced rapidly to perform their tasks, and once the work is done they need to disappear quickly. Therefore, in the situation regarding status-transition, the miRNAs needed by the transition tend to be highly expressed and should have rapid turnover. According to this hypothesis, because two embryo-derived cells (H9 and HEK293) have great potential of status-transition, they have low miRNA stability. In contrast, the human neural stem cell (NSC), although also an embryo-derived cell, may have lower potential of status-transition, and therefore, it does not need more miRNAs with higher stability. This distinct mode may be determined by the specific role of miRNAs in gene regulation. Because miRNAs negatively regulate target genes, the two types of molecules will be mutually exclusive. Normally, there exists a strong negative correlation between gene expression level and its tissue specificity [40]. This correlation also exists in miRNAs. Because of negative regulatory relationship between miRNAs and target genes, widely (highly) expressed genes do not tend to be regulated by highly (widely) expressed miRNAs. If the miRNA-target interaction needs a time mode of cooperation, they should correlate with each other in stability. Therefore, the distinct relationship between stability and expression level of miRNAs may be at least partly resulted from the distinct role of miRNAs in gene regulation.

### 3.3. The Effects of miRNA Up-/Downstream Molecules on miRNA Stability

In the above analysis, we revealed a negative correlation between miRNA stability and its expression level, a distinct mode of miRNA action that may be determined by the special role of miRNAs in the negative regulation of target genes. Therefore, it is reasonable to hypothesize that the stability of miRNAs has relationship with that of their targets. Although it has been previously reported that the stability of miRNAs and their targets can mutually affect each other [23] for specific miRNA-target cases, a global relationship between the stabilities of the two types of molecules remains unclear but is critically important for the understanding of miRNA functions in cellular processes. No biological molecules are isolated but interact with each other and have mutual effects. As one class of gene regulators, miRNAs regulate downstream targets and are regulated by upstream regulators as well, such as transcription factors. Here, we first investigated the relationship between the stability of miRNAs and that of their targets. Because the protein stability data of HEK293 cell is available, we focused on this part of analysis of this cell. To ensure the accuracy, we used the experimentally supported miRNA-target pairs. As a result, the stability of miRNAs shows a positive correlation with that of their targets (*R* = 0.1, *P* = 0.02). This result is consistent with the above analysis for the distinct relationship of miRNA stability and miRNA expression level. We next analyzed the relationship between the stability of miRNAs and that of their transcription factors (TFs). For accuracy, we focused on the experimentally supported TF-miRNA pairs. The result showed that there exists a significant negative correlation between the stability of miRNAs and that of TFs for the TF-activating-miRNA pairs (*R* = −0.30, *P* = 0.04), whereas the correlation for the TF-repressing-miRNA pairs tends to be positive but not statistically significant (*R* = 0.16, *P* = 0.50). Overall, these findings suggest that the stability of miRNAs was affected by their interacting partners.

### 3.4. Functional Analysis of Stable and Unstable miRNAs

**Figure 4:**
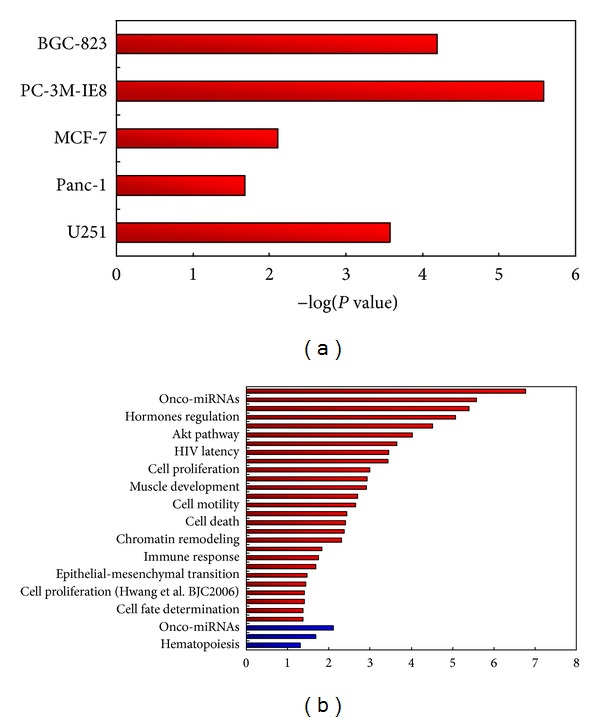
Functional analysis of miRNAs. (a) Enrichment of unstable miRNAs in oncogenic activity for five cancer cell lines. (b) Functional enrichment of unstable miRNAs (red bars) and stable miRNAs (blue bars) for the H9 cell.

The above analysis has revealed a distinct mode of miRNA stability, “rapid production, rapid turnover; slow production, slow turnover”. This mode may be dependent on the special role of miRNA functions in gene regulation. Thus, it is reasonable to speculate that there are differences in functions of stable and unstable miRNAs. We classified miRNAs into stable miRNAs (MSI > 0.5) and unstable miRNAs (MSI ≤ 0.5). Then, we performed functional analysis of these miRNAs using TAM [38], a tool for miRNA set enrichment analysis. The results indicated that miRNAs with rapid turnover in almost all of the cancer cell lines (5/6) are more enriched in oncogenic activity rather than tumor suppressing activity, suggesting that oncogenic power in these cell lines is mostly active (Figure 4(a)). The unstable miRNAs tend to implicate in more functions than the stable ones in most of the cell types. For example, in the human embryonic stem cell (H9), the unstable miRNAs are significantly enriched in 27 diverse functions, whereas the stable ones only enriched in three functions (Figure 4(b)). This suggests a great potential of status-transition of the human embryonic stem cell. Moreover, the mostly enriched function of unstable miRNAs in H9 cells is the function of “human embryonic stem cell (hESC) regulation” (*P* = 1.7 × 10^−7^), which is consistent with the characteristics of H9 cells. While in the neural stem cell (NSC), it is the stable but not the unstable miRNAs enriched in the function of “Human embryonic stem cell (hESC) regulation” (*P* = 0.003). This may explain why the NSC cells have greater miRNA stability than the other two embryo-derived H9 and HEK293 cells. The HEK293 cell is an embryo-derived cell but not stem cell, and its presented miRNAs are not associated with the function of “human embryonic stem cell (hESC) regulation.” The human smooth muscle cell (T/G HA-VSMC) has different patterns compared with other cells. It has the least presented miRNAs, and its stable and not the unstable miRNAs are enriched in more functions. The stable and unstable miRNAs of the smooth muscle cell tend to show related but different functions. For example, its unstable miRNAs are enriched in glucose metabolism, whereas the stable miRNAs are enriched in lipid metabolism. Together, the above results suggest that stable and unstable miRNAs have different biological functions, and therefore, the stability of miRNAs may play important roles in regulation of miRNA functions. 

### 3.5. miRNA Stability Elements

**Figure 5:**
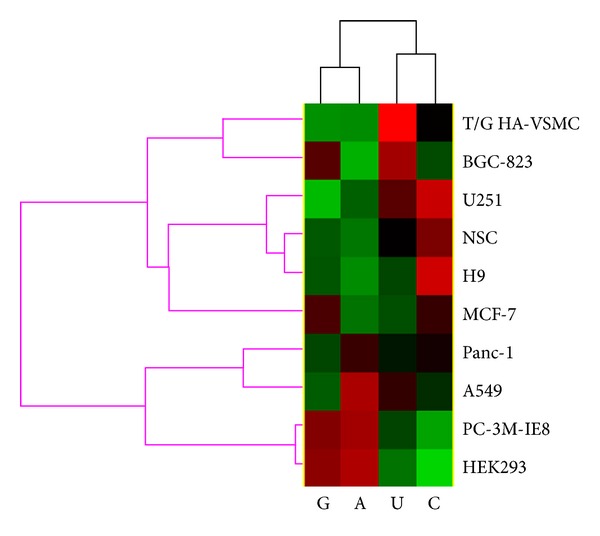
Correlation of miRNA stability (miRNA stability index, MSI) and nucleotide content (A, C, G, and U). Biclustering of the correlation coefficient was performed using Cluster software. The red color means a positive correlation, and the green color means a negative correlation.

A number of stability elements for biological molecules have been identified, including AU-rich elements at mRNA 3′UTR [41], number of introns, cellular location, and gene length [18]. Here, we investigated the roles of various biological elements in regulation of the stability of miRNA. We first investigated whether the stability of miRNAs is correlated with the nucleotide content of miRNAs. The result showed that miRNA stability is significantly correlated with the miRNA content of adenosine (A), cytidine (C), guanosine (G), and uridine (U) (Figure 5, Supplementary Table S3). Moreover, the correlation is dependent on cell types (Figure 5). For example, the stability of miRNAs and adenosine content show positive correlations in A549 (*R* = 0.18, *P* = 0.005), HEK293 (*R* = 0.18, *P* = 0.001), and PC-3 M-IE8 (*R* = 0.17, *P* = 0.005), whereas they show negative correlations in BGC-823 (*R* = −0.18, *P* = 0.02), H9 (*R* = −0.14, *P* = 0.005), and NSC (*R* = −0.12, *P* = 0.02). Moreover, the correlation is also dependent on nucleotide types. For example, in HEK293 cell, miRNA stability is positively correlated with A (*R* = 0.18, *P* = 0.001) and G (*R* = 0.15, *P* = 0.01) but negatively correlated with C (*R* = −0.22, *P* = 8.72 × 10^−5^) and U (*R* = −0.12, *P* = 0.03) content. Overall, A and G have similar pattern, which is opposite to that of C and U (Figure 5). However, unlike mRNAs and proteins, no significant correlation was found between the stability of miRNAs and their length. The reason may be that miRNAs are very small in length, and therefore, they do not depend too much on the materials and energy used to produce them. Furthermore, no correlation was found between miRNA stability and the predicted free energy of its precursor sequence. 

**Figure 6:**
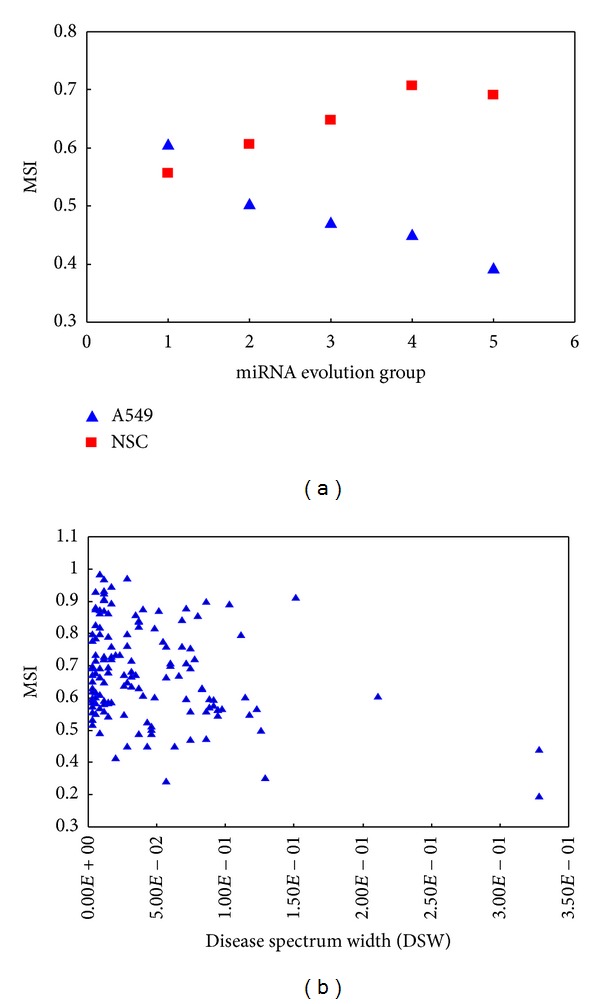
Correlation of miRNA stability (miRNA stability index, MSI) and miRNA evolution (a) and disease spectrum width (DSW) (b). For miRNA evolution, from group 1 to group 5, the miRNAs in these groups are miRNAs conserved in invertebrates, vertebrates, mammals, primates, and human, respectively.

We also observed a correlation between miRNA stability and its evolution (conservation). Similar to miRNA nucleotide content, the correlation between miRNA stability and miRNA evolution is also dependent on cell types. The correlation is negative in the lung cancer cell (A549) (*R* = −0.16, *P* = 0.12, Figure 6(a)) but positive in the neural stem cell (NSC) (*R* = 0.14, *P* = 0.008, Figure 6(a)) and the glioblastoma cell (U251) (*R* = 0.16, *P* = 0.01). miRNAs are not randomly located in the genome. It is known that a number of human miRNAs locate within the introns of protein-coding genes. Further analysis on the stability of intronic miRNAs and intergenic miRNAs revealed no difference between them. Moreover, no significant correlation was found between the stability of intronic miRNAs and that of their host genes.

Previous studies have shown that miRNAs, environmental factors (EFs), and human diseases have complex relationships, which are not random but show regular rules [11, 36]. We found that the stability of miRNAs is negatively correlated with the disease spectrum width (DSW) of miRNAs in the pancreas carcinoma cell (Panc-1) (Figure 6(b)). DSW of a miRNA is defined to measure its importance or chance in the development and progression of human diseases. This result suggests that miRNAs mostly implicated in human disease tend to have rapid turnover in the pancreas carcinoma cell. No statistically significant correlation between the stability and DSW of miRNAs was found in other cell types. According to the miREnvironment, a database for relationships of miRNA, EFs, and disease, MCF-7 is a popular cell line for the investigation of mutual effects between miRNAs and EFs. Here, we compared the stability of miRNAs that respond to EFs in MCF-7 with the stability of miRNAs that are not reported to respond to EFs. The result showed that the miRNAs that respond to EFs have lower stability (Supplementary Figure S2), which suggests that miRNAs with rapid turnover more likely tend to implicate in the response to EFs in the MCF-7 cell. 

## 4. Discussion

In this study, we systematically determined the genome-wide stability of miRNAs in ten human cell lines that cover a broad range of cell types and represent a diversity of cellular conditions. We then performed a comprehensive analysis to the data of miRNA stability. Although miRNAs are normally assumed to be very stable [26, 42, 43], we observed that miRNAs have a wide range of stability in individual cells. Our results indicated that a number of miRNAs show rapid turnover, which is consistent with recent studies on miRNA stability in mouse cells [27, 28]. Besides the significant diversity of miRNA stability in single cells, we further found that miRNA stability shows high dynamics across cell types. Different cell types show greatly diverse distributions of miRNA stability, suggesting that cells may have distinct profile of miRNA stability and cell types could have strong effects on miRNA stability. Therefore, miRNA stability may play important roles in determining cellular identity and functions. Strikingly, we revealed that miRNA stability is negatively correlated with its expression level. This novel finding is different from other biological molecules such as protein, mRNA, and noncoding transcript, which all show a positive correlation between the stability and expression level. This observation also revealed a distinct mode of miRNA stability, which we called “rapid production, rapid turnover and slow production, slow turnover.” This mode may be dependent on the specific regulatory mode of miRNAs. Because miRNAs mainly regulate gene expression in a negative manner, the highly expressed (also broadly expressed) miRNAs therefore tend to regulate narrowly expressed (also lowly expressed) genes, and vice versa. Therefore, miRNAs should have opposite pattern to target genes. In a previous study on plant mRNA stability, Narsai et al. found that miRNA target mRNAs are generally unstable [18]. Given that more high expression miRNAs were identified than low expression miRNAs at that time, this study gave clues that high expression miRNAs may have rapid turnover compared with low expression miRNAs. Although the exact reasons why the correlation between miRNA stability and its expression level is negative keep unknown, it is reasonable to propose that the miRNAs with “rapid production and rapid turnover” may mainly implicate in biological processes of cellular status-transition. When there is a need for these miRNAs in cellular status-transition, they will be produced rapidly for the purpose of status-transition. In support, an obvious tendency of negative correlation between miRNA stability and miRNA level was observed during the physiological process of light adaptation in mouse eyes. This suggests that during a process of in vivo physiology-transition, the miRNAs with high level do tend to degrade rapidly. In addition, functional analysis revealed significant differences between stable and unstable miRNAs.

Moreover, we globally revealed that miRNA stability is not isolated but affected by both miRNA downstream targets and upstream TFs. This finding suggests that miRNA stability plays roles in physiology and pathophysiology in the context of molecular networks, and therefore, future studies on miRNA stability should pay attention to miRNA networks. In future, intensive study on miRNA networks will shed exciting light on understanding the stability of miRNA and its impact on target gene expression. We also revealed that miRNA stability is associated with a number of miRNA elements, including nucleotide content, miRNA-associated disease spectrum width, and miRNA-associated environmental factors in specific cellular condition. All these findings indicate that miRNAs have distinct mode of stability, which is not isolated but highly related with other biological molecules and miRNA elements. 

In recent years, genome-wide techniques (e.g., microarrays) in particular the transcriptomes of miRNAs have been widely used to study the biological function of miRNAs. Our research in this study strongly suggested that besides miRNA transcriptome, miRNA stability is an indispensable dimension to figure out miRNA functions. One innovation of this study is that we investigated the stability of miRNAs in diverse human cell types, which allow us to not only investigate the diversity of miRNA stability in individual cells but also explore the dynamics of miRNA stability across different cellular context. More importantly, this study revealed a number of important novel findings including the negative correlation between miRNA stability and miRNA expression level, a surprisingly different pattern from current findings on other biological molecules. 

The process of miRNA biogenesis including miRNA degradation is very complicated, and a number of factors were reported to affect miRNA degradation [23]. For example, besides transcription, the process of precursor miRNAs to mature miRNAs also has an important role in maintaining miRNA abundance [23]. In addition, a number of proteins (e.g., AGO2) and enzymes (e.g., XRN1, RRP41, and PNPase) affect miRNA stability. Modification of miRNA precursors also has effects on miRNA stability [44]. However, most of the above factors are not considered in current genome-wide studies on miRNA stability and should be integrated into future studies. 

Taken together, although our knowledge about when, how, and why miRNA degradation occurs is still limited, we determined the miRNA stability of 10 types of human cells and revealed a number of biological insights. The data and findings of this study together presented us a global landscape for human miRNA stability, which greatly improved our understanding of miRNA stability and functions.

## Supplementary Material

Supplementary Figure S1: Relationships among the ten cells linked by significant correlation (A) and mapped on a 2-dimensional Euclidean space by multidimensional scaling (MDS) based on miRNA stability data (B). For the cell network (A), a red link means a significant positive correlation (P<=0.05) of miRNA stability between the two linked cell lines, whereas a blue link means a significant negative correlation (P<=0.05).
Supplementary Figure S2: Comparison of miRNA stability (miRNA stability index, MSI) between miRNAs that responde to environmental factor (EF, red bar) and miRNAs that are not reported to respond EF (nonEF, green bar) in the MCF-7 cell.

## Figures and Tables

**Table 1:** Summary of the ten human cell types used in this study and the result of correlation analysis of miRNA stability and miRNA expression level in these cells.

Cell	Cell description	Derived tissue	Correlation coefficient, *P* value
A549	Human lung adenocarcinoma epithelial cell line	Lung	−0.42, 1.37*e* − 12
BGC-823	Human gastric cancer cell line	Stomach	−0.50, 1.92*e* − 12
HEK293	Human embryonic kidney 293 cell line	Embryonic kidney	−0.33, 1.21*e* − 09
H9	Human embryonic stem cell line	Blastocyst	−0.07, 0.1474
PC-3M-IE8	Human prostate cancer cell line	Prostate	−0.14, 0.0202
T/G HA-VSMC	Human smooth muscle cell line from the normal aorta	Muscle	−0.33, 8.24*e* − 04
MCF-7	Human breast cancer cell line	Breast	−0.29, 2.35*E* − 06
NSC	Human neural stem cell	Embryonic brain	−0.22, 3.41*e* − 05
Panc-1	Human pancreas carcinoma cell line	Pancreas	−0.34, 7.83*e* − 10
U251	Human glioblastoma cell line	Brain	−0.44, 2.19*e* − 13
