# Supplementary figures and images for: Genome-Wide Analysis of Human MicroRNA Stability
Source: Biomed Res Int. 2013 Sep 28;2013:368975. doi: 10.1155/2013/368975 (PMC3804285; doi:10.1155/2013/368975)

# Supplementary Figure S1

A

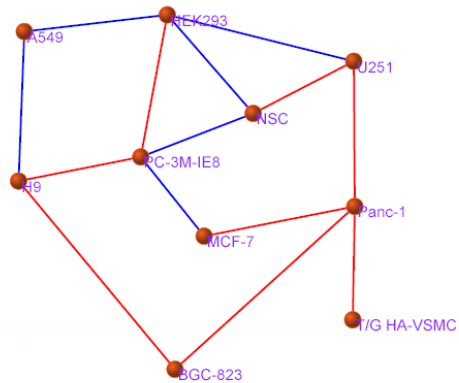

B

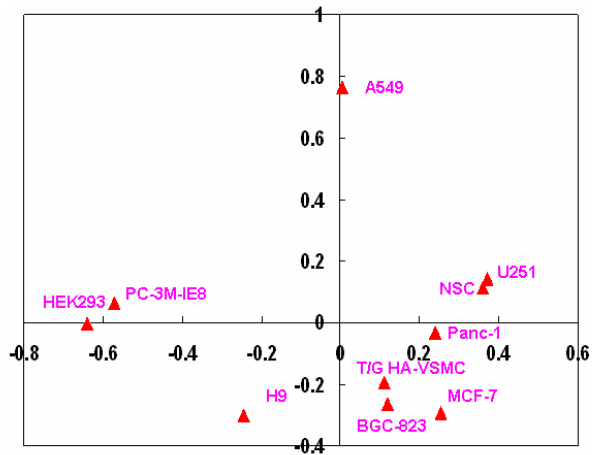

Supplementary Figure S2

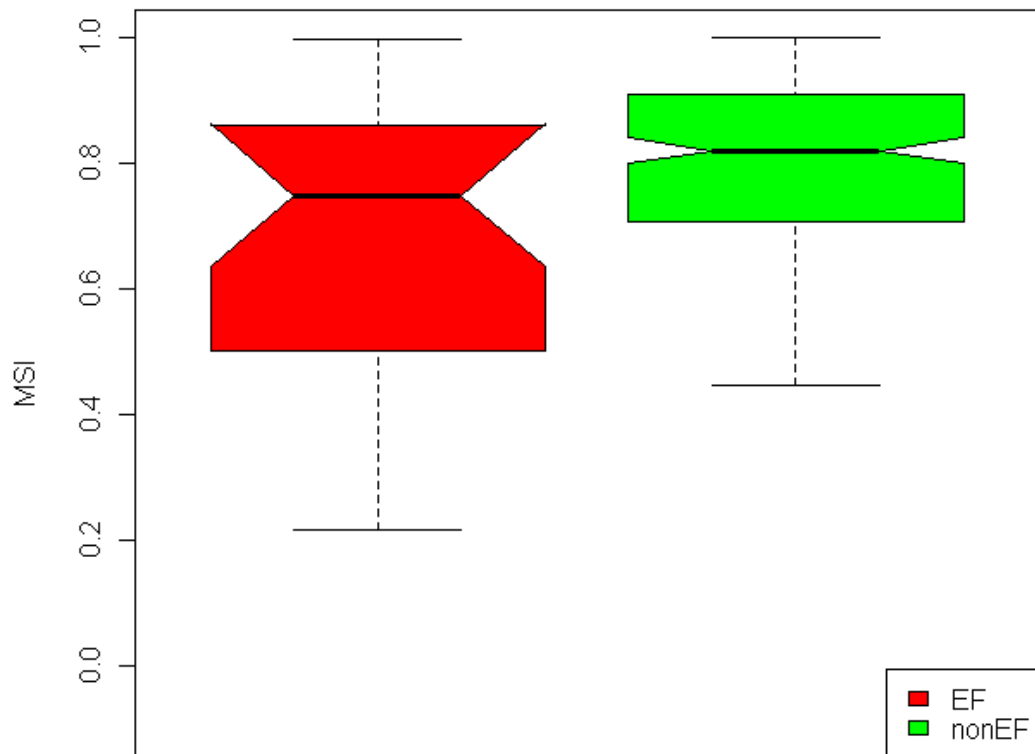

Supplement: Supplementary file 1 — Supplementary Figure S1: Relationships among the ten cells linked by significant correlation (A) and mapped on a 2-dimensional Euclidean space by multidimensional scaling (MDS) based on miRNA stability data (B). For the cell network (A), a red link means a significant positive correlation (P<=0.05) of miRNA stability between the two linked cell lines, whereas a blue link means a significant negative correlation (P<=0.05). Supplementary Figure S2: Comparison of miRNA stability (miRNA stability index, MSI) between miRNAs that responde to environmental factor (EF, red bar) and miRNAs that are not reported to respond EF (nonEF, green bar) in the MCF-7 cell. [file 368975.f1.pdf]
